# The Mechanical Properties and Energy Absorption of AuxHex Structures

**DOI:** 10.3390/ma17246073

**Published:** 2024-12-12

**Authors:** Robert Panowicz, Adam Jeschke, Tomasz Durejko, Marcin Zachman, Marcin Konarzewski

**Affiliations:** 1 Faculty of Mechanical Engineering, Military University of Technology, Gen. Kaliskiego Str., 00-908 Warsaw, Poland; adam.jeschke@wat.edu.pl (A.J.); marcin.konarzewski@wat.edu.pl (M.K.); 2 Faculty of Advanced Technologies and Chemistry, Military University of Technology, Gen. Kaliskiego Str., 00-908 Warsaw, Poland; tomasz.durejko@wat.edu.pl (T.D.); marcin.zachman@wat.edu.pl (M.Z.)

**Keywords:** 3D printing, cellular, structures, polymers

## Abstract

Based on a combination of hexagonal honeycomb and re-entrant honeycomb cells, the concept of novel hybrid cell structures was developed. Experimental studies and numerical analyses of the behaviour of the analysed structures under in-plane compression in two compression directions were carried out. Explicit finite element analyses with an explicit integration scheme, incorporating plastic deformation and ductile damage evolution models, were employed to analyse the entire deformation process, including plastic and damage stages. Good agreement was obtained between the results of the numerical analyses and the experimental studies.

## 1. Introduction

The development of 3D printing capabilities for polymeric materials [1,2] and metals [3,4,5] has made it possible to produce materials with both positive and negative Poisson’s ratio. The 3D printing makes it possible to reflect and investigate various structures found in nature [6,7,8]. With the ability to control the macroscopic properties of the printed structures, they can find applications in the space, aerospace, automotive, or biomedical industries. Density, or rather relative density, is usually considered to be the most significant design factor in the design of cellular structures. In most cases, higher mechanical properties correspond to higher relative density. The use of 3D printing enables not only the control of Young’s modulus, density, or shear modulus [9,10], but also fracture toughness [11,12], indentation resistance [13,14,15], thermal insulation [16], energy absorption capacity [1,5], or porosity with strain [17]. The ability to control such a wide range of properties has led to a rapid increase in interest in various types of both 2D [5,18,19] and 3D structures [1,2,20,21]. The structures have been investigated through numerical and theoretical analysis or experimental tests. Some of the first analyses of 2D hexagonal and re-entry auxetic structures were presented by Gibson and Ashby in their book [19] and in Masters and Evans’ paper [22]. These authors used the energy method to obtain Young’s modulus and Poisson’s ratio. On the other hand, Wang et al. used a strain-based expansion homogenisation method to determine the analytical relationships of the basic mechanical parameters of the structures [23]. Liu et al. studied in-plane crushing behaviours of honeycomb with Poisson’s ratio varying from positive values through zero to negative values [24]. They identified the three types of deformation occurring as a function of various crushing speeds as well as the velocity ranges in which the deformation types occur. The study was performed for structures with Poisson’s ratio from −3.3 to 3.3. Whereas Dong et al., studying auxetic re-entrant honeycombs structures characterised by different wall thicknesses, identified additional deformation types [18]. The study shows that for structures with thicker walls, the elastic strain energy can be neglected in the absorbed energy balance. Shao et al. analysed the deformation and energy absorption process of gradient structures occurring due to static and dynamic loading [25]. The studied auxetic re-entrant honeycomb gradient structures in each layer differed in wall thickness. In the range of low loading rates, the behaviour of the analysed gradient structures is similar. For high compression velocities, the location of the load application is important. More energy is absorbed by a structure loaded on the side having thicker walls. Out-of-plane and in-plane compression of hexagonal re-entrant honeycomb and other structures were investigated by Alomarah et al. [26]. When honeycomb structures are compressed out-of-plane, they have a longer plateau than re-entrant honeycomb structures, and the re-entrant structures have a higher Young’s modulus. Shen et al. built a hierarchical model based on the re-entrant honeycomb structure [27]. They compared the effect of modification on negative Poisson’s ratio and effective Young’s modulus. The implemented modifications allowed us to enhance the elastic modulus and maintain the negative Poisson’s ratio in a wide range. Schwerdtfeger et al. proposed a 3D structure on the basis of re-entrant honeycomb structure [28], which has also been analysed in other works using both experimental studies, numerical analyses, and theoretical considerations [20,29]. Broccolo et al. combined hexagonal honeycomb and re-entrant honeycomb cells to obtain a zero Poisson’s ratio structure appearing in the literature as AuxHex [30]. The ultimate compressive strength of the AuxHex is more than twice that of the honeycomb, even if the value of their relative densities is very similar. This structure was extended by Guo et al. to the 3D case [31]. Compared to the AuxHex 2D structure, the AuxHex 3D structure has better energy absorption performances under quasi-static and dynamic compression.

Nowadays, one of the most effective fabrication methods of polymer cellular structures is the fused filament fabrication (FFF) method. FFF is defined in an ASTM standard as “a material extrusion process used to make thermoplastic parts through heated extrusion and deposition of materials layer by layer” [32]. FFF parts can replace traditionally produced components in many areas, such as the automotive, aviation, or medical industries. However, there is still a lack of industry standards and knowledge about material behaviour under different loading conditions, including dynamic compression. The FFF method enables manufacturing not only 3D models but also structural parts using high-performance materials such as polyetherimides (PEI) or polyetheretherketones (PEEK) [33]. One of these materials is polyetherimide, known as ULTEM 9085, which is characterised by high mechanical strength (approx. 70 MPa of strength and 5.3% elongation break in tensile test) and high impact (88 J/m for the notched sample and 650 J/m for a sample without a notch), high heat (even 180 °C), flame, and all kinds of solvent (synthetic or natural) resistance [34]. ULTEM 9085 is interesting to use for highly responsible applications in the aerospace, automotive, and military sectors [35]. Moreover, ULTEM 9085 offers easy shaping during 3D printing with high dimensional stability.

The properties of AuxHex structures led us to investigate the properties of structures that are a combination of hexagonal honeycomb and re-entrant honeycomb cells. The initial section of the article introduces the AuxHex structures under investigation, detailing the fabrication process employing 3D printing via the fused filament fabrication (FFF) technique. Subsequently, the methodology of the experimental research is outlined. This includes the determination of the material properties of ULTEM 9085, utilised in the manufacturing of the structures, followed by compression testing of the fabricated AuxHex structures. Structural parameters, particularly those related to energy absorption capabilities, are described and quantified through experimental investigations. The concluding section of the article presents the outcomes of numerical analyses conducted using the finite element method (FEM) and compares these findings with the experimental results.

## 2. Materials and Methods

### 2.1. Materials and Samples Fabrication

**Figure 1:**
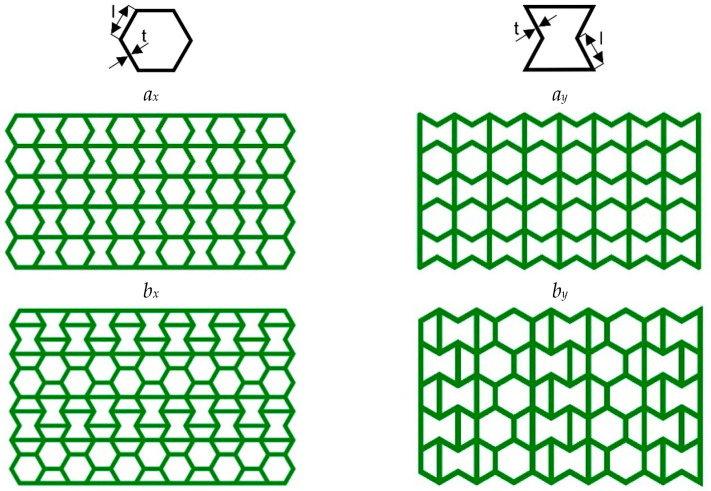
Basic cells (t = 1.05 mm, l = 4 mm) and analysed structures. *x* and *y* index indicates the compression direction defined concerning the structure.

The high-performance ULTEM 9085 polyetherimide in filament form (Thermax TM PEI produced by 3DXTECH company, Grand Rapids, MI, USA), which combines excellent mechanical properties, chemical resistance, and exceptional dimensional stability, was used. Moreover, it meets multiple transportation industry standards for flame, smoke, and toxicity requirements [36]. The INTAMSYS FUNMAT HT Enhanced machine was used to manufacture cellular structures (Shanghai, China). For this research, the AuxHex (a combination of hexagonal honeycomb and re-entrant honeycomb cells, Figure 1) structures were modelled using commercial CAD software (Autodesk Inventor v. 2024) and saved as STL files. In the next step, prepared STL objects were sliced in InstamSuite 4.0 software to a given layer thickness. Furthermore, the printing parameters were added (Table 1), and the tool path was generated and saved as a Gcode file. After adjusting the 3D printer setup (adjusting the building platform, installing the material, etc.), both cellular and tensile samples were fabricated.

Finally, three samples for each geometrical variant were manufactured. The samples were analysed using the CT method and compared with STL models. The tomographic inspection was carried out using the Nikon/METRIS XT H 225 ST apparatus (Tokyo, Japan), which is a micro-focus X-ray system designed for industrial and scientific applications. The ULTEM 9085 cellular AuxHex samples were scanned with parameters shown in Table 2 and were next reconstructed in VG studio MAX 3.2 software. The obtained dimensions are presented in Table 3.

The dimensional accuracy of produced samples was typical for the FFF method. The average length/width and height were approx. 0.3 mm and 0.2 mm lower than for STL models, respectively. However, the average wall thickness was 1.05 for all cellular configuration variants at an accuracy of approx. 0.05 mm compared with the STL model (Table 3).

### 2.2. Experimental Investigation

For the tests, it was decided to use the ULTEM 9085 material characterised by high strength (86 MPa) and a Young’s modulus (2230 MPa) at a heat deflection temperature of 153 °C [32]. This material also meets the rigorous requirements of the aviation industry.

**Figure 2:**
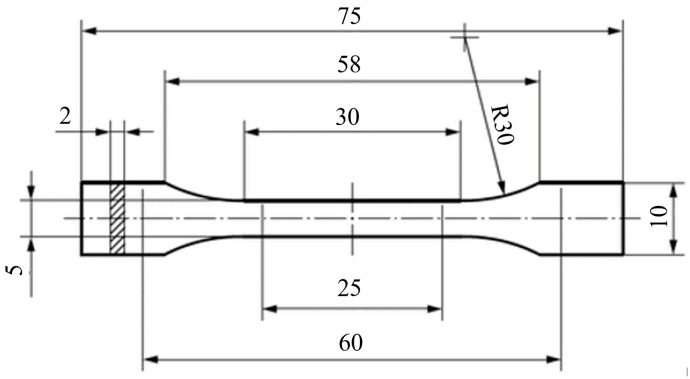
Reduced size 1BA sample (Unit: mm).

Firstly, tensile tests were carried out to determine the basic material parameters to be used as input for the subsequent numerical models. The tests were carried out in accordance with the guidelines of ISO-527 [37]. Due to the difficulties in making the standard-size specimens, it was decided to use the reduced type 1BA specimens, which were prepared using the FFF (fused filament fabrication) 3D printing technique (Figure 2). The manufacturing process parameters for these samples were analogous to those used in the structures manufacturing and are presented in Table 1. The layer thickness was equal to 0.1 mm.

**Figure 3:**
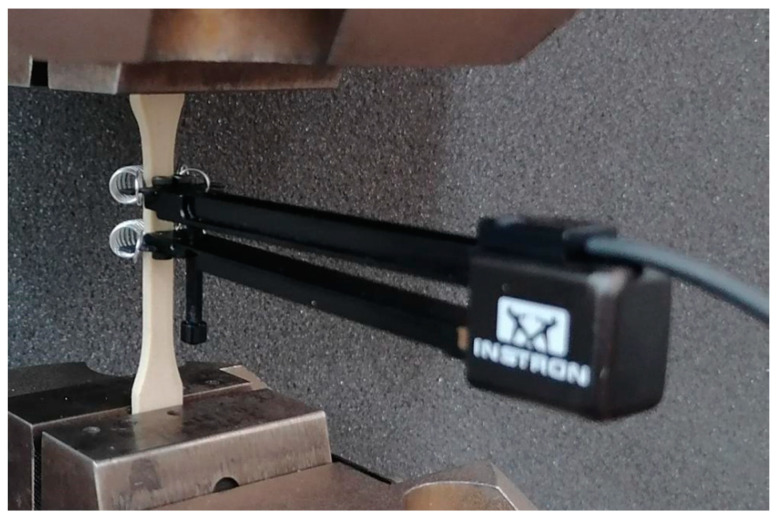
Sample mounted on the test stand with visible extensometer.

Tensile tests were conducted using an Instron 8862 electromechanical testing machine (Norwood, MA, USA), which has a maximum displacement range of ±300 mm and a maximum crosshead speed of 300 mm/min (Figure 3). A 5 kN load cell was utilised to measure the force. Three samples were tested. The article later presents the results of the repeatability test values.

The tests were performed in accordance with ISO 527-1 at a travel speed of 5 mm/min. Following the standard, the tests are conducted utilising dog bone-shaped specimens at a consistent deformation rate. The strain rate during the tests was constant and equal to 3.33 × 10^−2^ 1/s. The force and position of the crosshead were recorded at a frequency of 50 Hz. The strain of the specimens was determined using an extensometer. Tensile stress was calculated as the loading force divided by the initial cross-sectional area of the tested specimen. The highest stress and the elongation at the point of sample breakage are known as tensile strength and gauge length deformation at break, respectively (Figure 3). It should be noted that any transverse deformations of the sample during the test are not considered.

**Figure 4:**
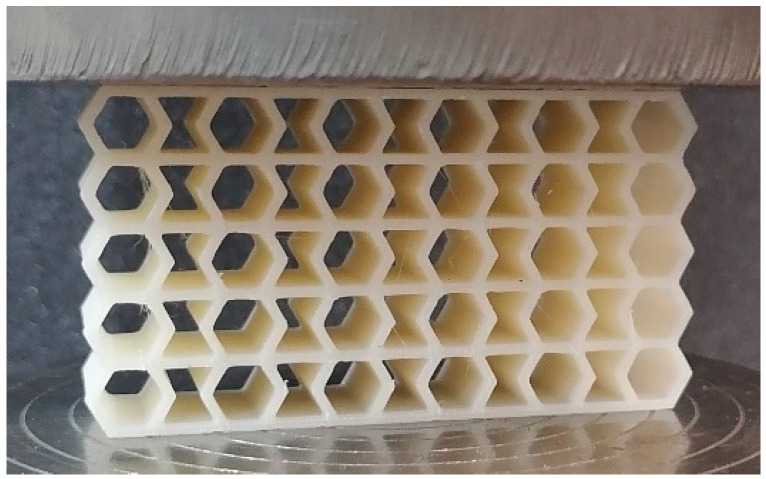
Structure mounted on the test stand.

In the next step, compression tests were carried out to test the structures (Figure 4). Analogous to the tensile tests, an Instron 8862 testing machine was used, but a 100 kN load cell was used due to the generally higher forces occurring in this type of test than in the tensile tests. Five samples of every structure were tested.

The tests were carried out in accordance with the guidelines of the ISO 844 standard [38], which states that the compression test should be carried out until the specimen thickness is reduced to 85% of the original thickness. Due to the limitations of the 100 kN load cell used in our case, and based on the preliminary tests, it was originally intended to carry out tests up to 80% of the original sample thickness. Unfortunately, in the case of some structures, this value was too high, and the forces obtained would be above the range of the load cell. In such instances, the test was stopped at 70% of the original thickness. In order to maintain a consistent appearance of the results presented, the strain on all graphs was also limited to 70% in all cases considered.

The second parameter specified by the standard is the compression rate, which should be as close as possible to 10% of the original specimen thickness per minute [38]. In the study presented here, all examined structures have a thickness of 20 mm. For this reason, a constant and universal compression rate of 2 mm per minute was chosen.

The testing machine recorded both force and crosshead displacement at a frequency of 50 Hz. To ensure the accuracy of the crosshead displacement measurements, a motion tracking system was employed. A 15 × 15 mm marker was placed on the crosshead, and the trials were recorded using a camera with a resolution of 1920 × 1080 pixels at a rate of 50 frames per second, which is consistent with the force recording rate. Following the trials, the recordings were imported into TEMA software version 3.0-024 (Image Systems AB), enabling tracking of the positional change of the aforementioned marker over time. The values obtained were compared with those obtained directly from the machine, and no significant differences were observed.

## 3. Parameters Characterizing the Structure

**Figure 5:**
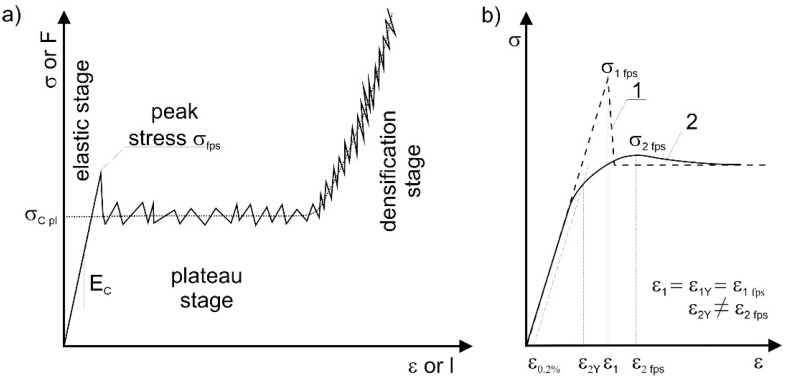
Cellular stress–strain curve; (**a**) brittle material; (**b**) initial range of the stress–strain curve with a distinct (curve 1) and without a distinct (curve 2) yield stress. The Y and fps indices denote the yield and the first peak, respectively; σ*_C pl_* is plateau stress.

**Figure 6:**
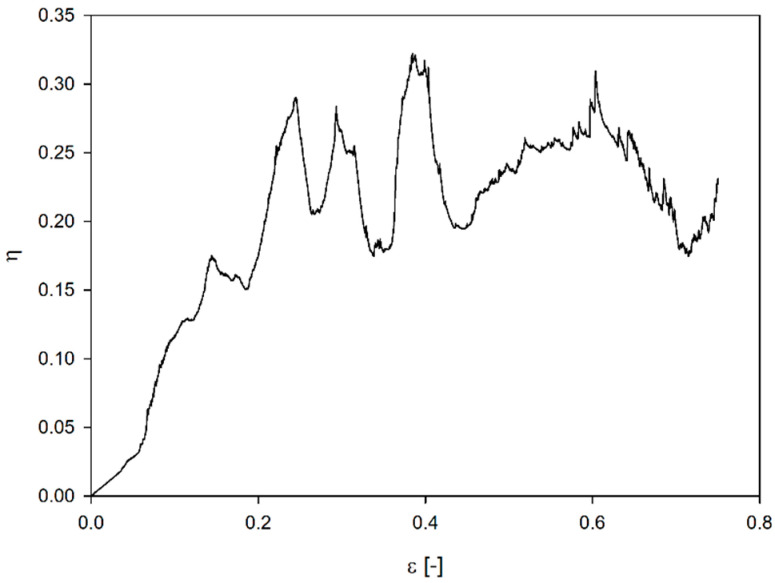
Energy absorption efficiency.

The stress–strain curve of cellular structures and various types of foams has a characteristic course (Figure 5a). In the initial, elastic range, the curve is defined by the Young’s modulus of the structure (*E_c_*). In the range of larger deformations, depending on the material’s properties (ductile, brittle) from which the structures were made and the type of structure, there are certain differences in the individual stress–strain curves. In some cases, the strain (ε_2p_) corresponding to the first stress peak (σ*_fps_*) (force) is significantly different from the strain (ε_2Y)_ corresponding to the yield strength (Figure 5b). Sometimes, changes in the values of the force recorded during the tests (brittle or ductile–brittle matter) also prevent an accurate or unambiguous determination of the densification strain (Figure 6) [18].

(1) $${\left.\frac{d\eta \left(\varepsilon \right)}{d\varepsilon }\right|}_{\varepsilon =\varepsilon_{d}}=0$$

where

(2) $$\eta \left(\varepsilon \right)=\frac{\int_{0}^{\varepsilon }\sigma \left(\tau \right)d\tau }{{\left.\sigma \left(\tau \right)\right|}_{\tau =\varepsilon }}$$

is energy absorption efficiency and ε_d_ is densification strain.

There are different approaches to the problems presented in the articles [18,39,40,41]. After analysing the different solutions, we propose that in the case of test results described by a curve similar to the one shown in Figure 2, the Yield strength and strain ε_2Y_ should be assumed as the plastic flow strain for materials with an indistinct yield point. On the other hand, to determine the densification strain, we propose using one of the parameters related to energy. It can be either total energy absorbed (EA), specific energy absorption (SEA), or energy related to a unit volume (EAV), written as follows:

– Total energy absorption (EA) (3) [39] is the total energy absorbed during the compression process, calculated as follows:

(3) $$EA=\int_{0}^{l}F\left(x\right)dx$$

where *F(x*) is compression force and *l* is compression distance;

– Specific energy absorption (SEA) (4) [41] is EA related to the mass of the energy absorber, which enables a simple comparison of the different solutions, calculated as follows:

(4) $$SEA=\frac{EA}{m}$$

where *m* is mass of the energy absorber.

– Total energy absorption (EAV) (5) is related to a unit of volume [39], calculated as follows:

(5) $$EAV=\int_{0}^{\varepsilon }\sigma \left(\tau \right)d\tau$$

In the rest of the article, EA is used to determine densification strain.

**Figure 7:**
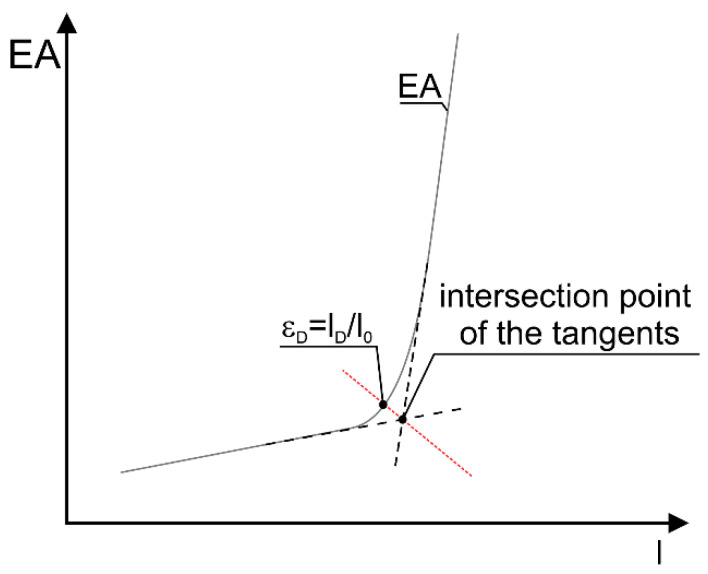
Schematic diagram of the *EA*−*l* curve with the method of determining ε_D_; l_0_ is the initial height of the sample.

The above-mentioned parameters (EA, SEA, and EAV) depend almost linearly on the strain or displacement in the plateau range as well as in the densification range. Therefore, the densification strain will be defined as the strain value corresponding to the projection of the intersection of the tangents in the plateau range and in the densification range on the EA curve (Figure 7).

This will allow us to determine the plateau stress (7) [39], as well as the remaining parameters in the useful range, calculated as follows:

(6) $$\sigma_{c\;pl}=\frac{1}{\varepsilon_{d}-\varepsilon_{Y}}\int_{\varepsilon_{Y}}^{\varepsilon_{d}}\sigma \left(\tau \right)d\tau$$

where ε_Y_ is strain at yield and ε_d_ is densification strain.

A number of the listed parameters according to the classical theory of cellular solids depend on the relative density ($\rho^{*}=\rho_{c}/\rho_{s}$) [19], calculated as follows:

(7) $$\frac{E_{c}}{E_{s}}=a_{E}{\left(\rho^{*}\right)}^{n_{E}}$$

(8) $$\frac{\sigma_{c\;pl}}{\sigma_{sY}}=a_{pl}{\left(\rho^{*}\right)}^{n_{pl}}$$

(9) $$\frac{EA}{\sigma_{sY}}=a_{EA}{\left(\rho^{*}\right)}^{n_{SEA}}$$

where *ρ_s_* is the density of the parent material, *ρ_c_* is the density of the structure, the subscripts *c* and *s* refer to the properties of the structure and parent material, respectively, and *a* and *n* are structure-specific constants while subscripts (*E*, *pl*, and *SEA*) refer to the parameter in question.

If n_pl_ is equal to 1, cellular exhibits a standard stretching-dominated mode, and when n_pl_ is equal 1.5, it exhibits a standard bending-dominated deformation mode [19].

In contrast, densification strain depends linearly on relative density, written as follows:

(10) $$\varepsilon_{d}=1-a_{d}\left(\rho^{*}/\rho_{s}\right)$$

where $a_{d}$ is material parameter.

## 4. Results

### 4.1. Tensile Test

**Figure 8:**
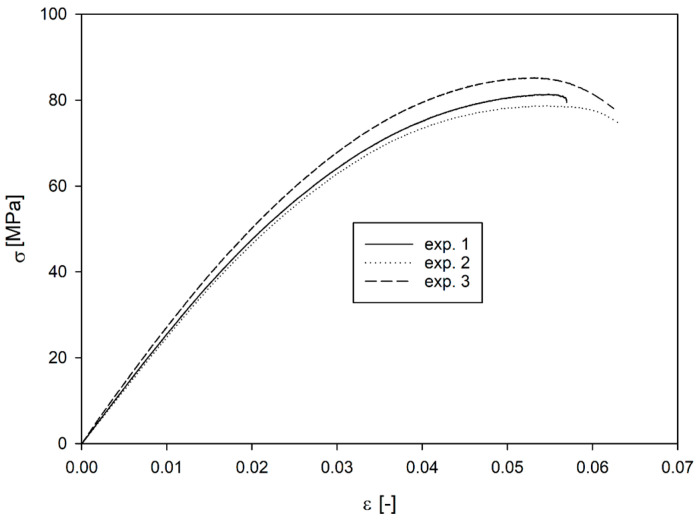
Engineering stress vs. engineering strain for ULTEM during tensile test.

Figure 8 shows the engineering stress vs. engineering strain curves for tested specimens. The material behaves similarly to brittle materials, although the slope of the curve is more like that of ductile materials. The average strain at fracture is 0.061, and the maximum stress is 81.7 MPa. The Young’s modulus, determined from the slope of the curve in its linear section, is 2624 MPa. Table 4 shows a summary of the obtained values. The Young’s modulus of the printed samples was almost 17% higher, and their strength was slightly lower (5%) than the reference data presented in the introduction.

### 4.2. Compression Test

**Figure 9:**
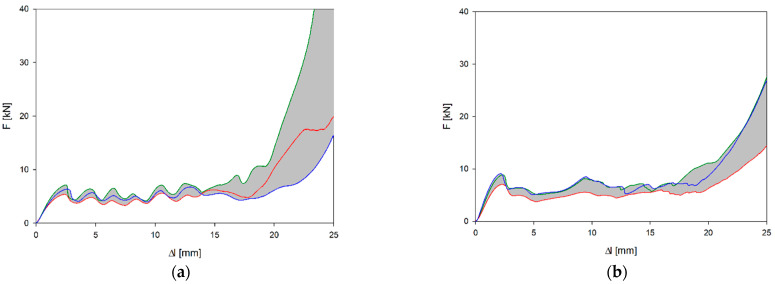
The force vs. change in length curves with the area of variation marked in grey for the (**a**) *a_x_* structure and (**b**) *b_x_* structure.

Figure 9a,b present the results of experimental studies in the form of the F-Δl dependencies of two selected structures marked in accordance with Figure 1. Table 4 contains the parameters characterising the structures determined on the basis of experimental studies and the dependencies presented above.

A high repeatability of the results of experimental studies of the structures is visible. The smallest differences occur in the useful range. In the case of the *a_x_* structure, waves corresponding to the folding of individual cellular layers are visible in the plateau range. Apart from peak stress, in the case of the *b_x_* structure, this course is much smoother. The *a_x_* and *a_y_* structures differ significantly in their parameters, while the differences in the values of various parameters of the *b_x_* and *b_y_* structures are relatively small (Table 5).

The relative difference in stiffness between *a_x_* and *a_y_* structures is more than 30%, while for b-type structures the difference is less than 10%. Similar percentage differences are found for plateau strain and specific energy absorption. The amount of energy absorbed by *a_x_* and *b_x_* structures is comparable. In contrast, the greatest differences in both relative and absolute value occur when comparing *a_y_* and *b_y_* structures. The *a_y_* structure absorbed only 35.36 J on average. In relation to the energy absorbed by the *a_x_* structure, this represents just under 33% of the value, while the *b_y_* structure absorbed 73.5 J (62.55% in relation to the *b_x_* structure).

## 5. Numerical Modelling

The numerical analyses were carried out using the commercial Ls-Dyna software version R12 (Ansys), containing a finite element (FE) method implementation for the analysis of dynamic phenomena [42].

**Figure 10:**
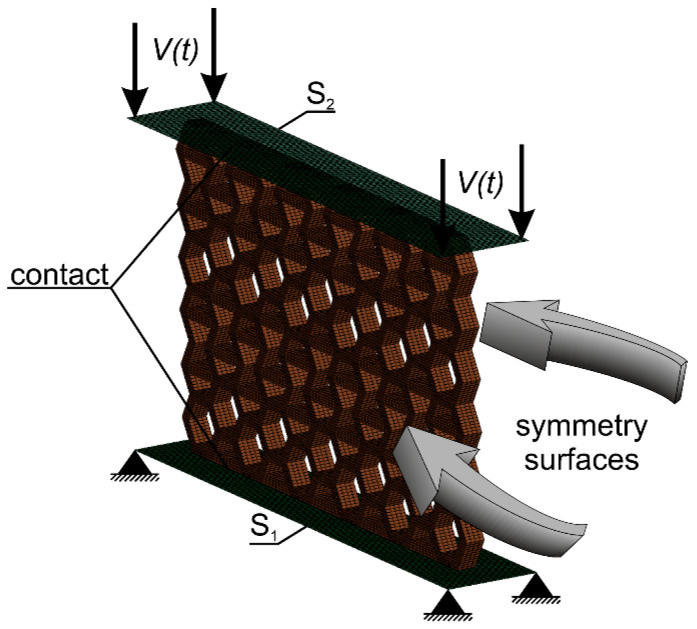
Numerical model of the experimental study.

The FE model replicated the experimental test conditions. The structure was placed between two non-deformable surfaces, S_1_ and S_2_ (Figure 10). The lower surface S_1_ corresponds to the table of the testing machine, while the upper surface S_2_ corresponds to the crosshead. Therefore, the upper surface S_2_ moved with a given velocity V, while the lower surface S_1_ was stationary. The interaction between the surfaces and the structure was realised by means of contact based on a penalty function [42]. The coefficient of friction between the 3D structures and the table or the crosshead was set to 0.2, while the friction coefficient between the structure elements was set to 0.65. These values were derived from the literature data. Subsequently, preliminary analyses were performed, during which the friction coefficient values were adjusted to ensure alignment with the conditions observed in the experimental studies.

In order to avoid wave phenomena associated with sample loading and manifested in disturbances (oscillations) of the measured quantities, a time-varying, increasing crosshead velocity was adopted (11), calculated as follows [43]:

(11) $$V\left(t\right)=\frac{\pi }{\pi -2}\frac{\Delta l}{T}\left[1-cos\left(\frac{\pi }{2T}t\right)\right]$$

where *t* is time and Δ*l* is maximum crosshead displacement occurring at time *T*.

The analyses used fully integrated solid elements, which provide an accurate description also for elements with poor aspect ratio [42]. It should be noted that the use of fully integrated solid elements eliminates the possibility of zero energy modes, called hourglassing modes [42].

The average element dimension was 0.25 mm, which is consistent with other similar works in this area [5,20,26], as well as with the mesh sensitivity analysis carried out. In order to reduce computation time, only a 2 mm thick layer was analysed by assuming symmetry conditions on the outer surfaces of the layer (Figure 9). The resulting contact force values were later scaled to correspond to the real values recorded during experimental tests.

Due to the complex, heterogeneous internal texture of structures made by 3D printing, there are difficulties in selecting a material model that correctly describes its behaviour. The constitutive model used should take into account not only the fracture process but also the softening caused by the development and coalescence of voids or cracks [44,45]. In such a case, the true stress is determined by Equation (12), calculated as follows:

(12) $$\sigma_{true}=\frac{F}{A-A_{voids}}$$

where σ_true_ is true stress, *F* is measured force, *A* is all specimen cross-sectional area with voids area (*A_voids_*).

Defining the damage parameter or function of many parameters *D* as:

(13) $$D=\frac{A_{voids}}{A}$$

Equation (8) can be transformed to the known form (10) from the continuum damage mechanics, calculated as follows [46]:

(14) $$\sigma =\frac{F}{A}=\sigma_{true}\left(1-D\right).$$

**Figure 11:**
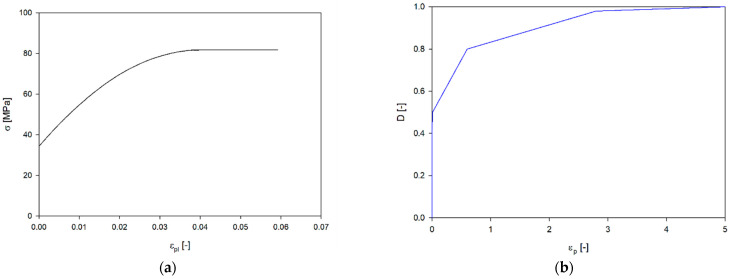
Curves used in the numerical analyses (**a**) the plastic true stress–strain curve; (**b**) material softening curve.

In general, the determination of the evolution process of the damage *D* requires a number of additional studies [47,48]. In [44], a more straightforward approach was proposed for printed materials. Tabacu and Ducu assumed a linear stress–strain relationship above the strain corresponding to the value of the ultimate tensile strength. In this work, this approach was also used, assuming the failure strain equal to the average failure strain of the samples (Table 2) (Figure 11a). On the other hand, the material failure process caused by the formation and development of voids was modelled by introducing a curve corresponding to the parameter D dependent on the plastic strain.

For this purpose, elasto-viscoplastic material models (*MAT_PLASTICITY_WITH_DAMAGE) with damage and softening were selected for numerical analyses. The stress–strain curve was described by a multilinear model with points of densification in the region of inflection (Figure 11a). The material damage (softening) curve was adopted analogously to the work of [5] (Figure 11b). The last segment of the curve results from the observation of the fracture process of the compressed structures. In the areas of plastic hinge, only partial destruction of the material while maintaining continuity of the remaining part. Fracture of the material occurs on the tension side, while the continuity of the material is maintained on the compression side.

Analyses of dynamic phenomena using FE method are related to the numerical solution of the following matrix equilibrium Equation (15):

(15) $$M\ddot{u}+C\dot{u}+Ku=F$$

where *M* is the mass matrix, *K* is the stiffness matrix, *C* is the damping matrix, *F* is the load vector, and *u* is the displacement vector.

The time derivatives appearing in Equation (15) are replaced by a finite difference approximation. Due to the second order of accuracy and the simple calculation scheme, the explicit central difference method is frequently used, written as follows in Equation (16):

(16) $$\begin{array}{c} {\ddot{u}}_{t}=\frac{1}{{∆t}^{2}}\left(u_{t+∆t}-2u_{t}+u_{t-∆t}\right),\\ {\dot{u}}_{t}=\frac{1}{2∆t}\left(u_{t+∆t}-u_{t-∆t}\right). \end{array}$$

Using (15) and (16), the displacement of nodes at time t + Δt can be expressed by the following Equation (17):

(17) $$\left(\frac{M}{{∆t}^{2}}+\frac{C}{2∆t}\right)u_{t+∆t}=F-\left(K-\frac{2M}{{∆t}^{2}}\right)u_{t}-\left(\frac{M}{{∆t}^{2}}-\frac{C}{2∆t}\right)u_{t-∆t}$$

The matrices *M* and *C* are diagonal, so determining the inverse matrix to them is trivial, simplifying the displacement determination algorithm considerably.

Two main problems occur in numerical analyses of dynamic phenomena that use an explicit time integration scheme. The first is the occurrence of unphysical oscillations associated with a given computational scheme and the physical phenomenon being analysed. To overcome their influence, artificial viscosity is used (18) [49,50]:

(18) $$q=\left\{\begin{array}{cc} C_{0}\rho L^{2}{\left(\frac{ds}{dt}\right)}^{2}+C_{L}\rho La\left|\frac{ds}{dt}\right|, & \frac{ds}{dt}<0\\ 0, & \frac{ds}{dt}\geq 0 \end{array}\right.$$

where *L* is characteristic mesh size, *ds*/*dt* is strain rate in the direction of acceleration, a is local sound speed, ρ is local density, and *C*_0_, *C_L_* are coefficients.

The calculation of *ds/dt* is associated with a large computational effort, so this quantity is replaced by the strain rate. The first term on the right-hand side of the Equation (18) is called von Neumann or quadratic artificial viscosity and is responsible for the damping of oscillations for which the square of the strain rate assumes large values. In contrast, the second term is responsible for the damping of oscillations with larger rise times.

The artificial viscosity *q* (19) is added to the pressure by modifying its value, written as follows:

(19) $$p_{cor}=p+q$$

**Figure 12:**
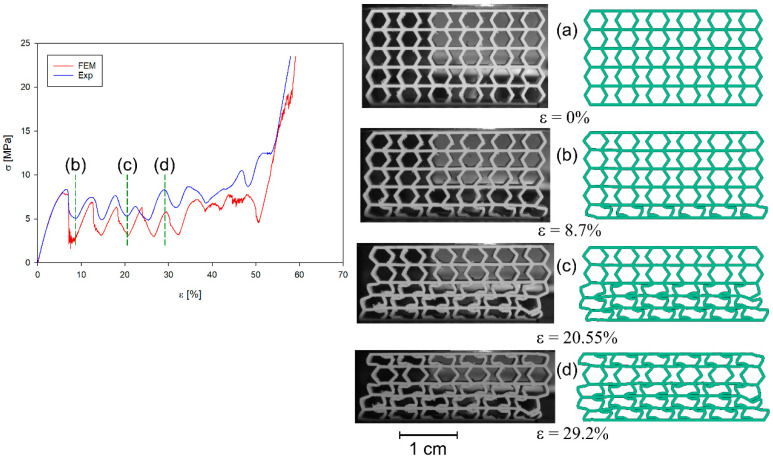
Compression stress–strain curve of experimental and FE analysis results for *a_x_* structure (**left**) and corresponding deformation behaviours at selected compression strains (**right**).

**Figure 13:**
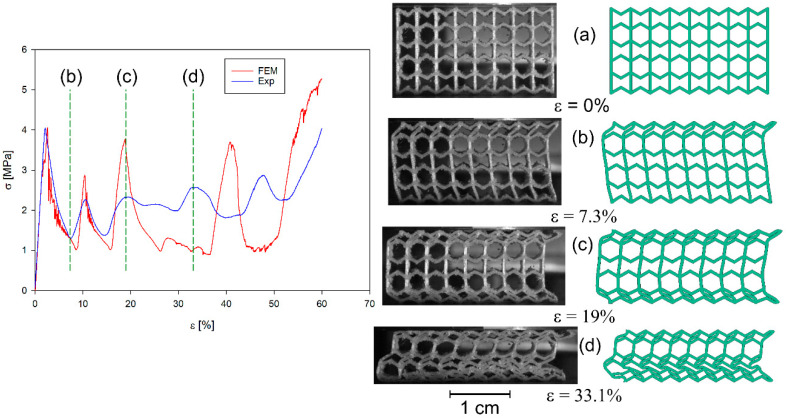
Compression stress–strain curve of experimental and FE analysis results for *a_y_* structure (**left**) and corresponding deformation behaviours at selected compression strains (**right**).

**Figure 14:**
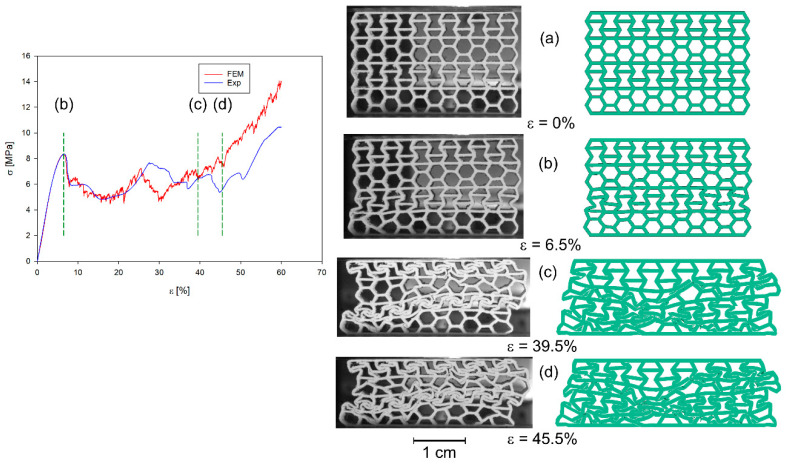
Compression stress–strain curve of experimental and FE analysis results for *b_x_* structure (**left**) and corresponding deformation behaviours at selected compression strains (**right**).

The increase in crosshead speed (11) plays an important role in the initial phase of the compression process of the structure, in terms of neutralising unwanted oscillations. Whereas the artificial viscosity (19) influences unwanted oscillations when the fragmentation process takes place and the released waves propagate from the fracture place [51], which can be seen in the form of jumps in the *F* − Δ*L* (σ − ε) presented later in this article (Figure 11, Figure 12, Figure 13 and Figure 14).

The second problem associated with the numerical analyses is the conditional stability of explicit time integration schemes. In the case of the explicit central difference method and the presence of the artificial viscosity, the condition on the time step takes the form of Equation (20), written as follows [42]:

(20) $$∆t=C_{CFL}\frac{L}{Q+\sqrt{Q^{2}+c^{2}}},$$

(21) $$Q=\left\{\begin{array}{cc} C_{0}L\left|\dot{\varepsilon }\right|+C_{L}c, & \dot{\varepsilon }<0\\ 0, & \dot{\varepsilon }\geq 0 \end{array}\right.$$

where *c* is adiabatic sound speed, and *C_CFL_ <* 1 is Courant–Friedrichs–Lewy number.

## 6. Discussion

### 6.1. Comparison of the Experimental Results with the Results of the Numerical Analyses

**Figure 15:**
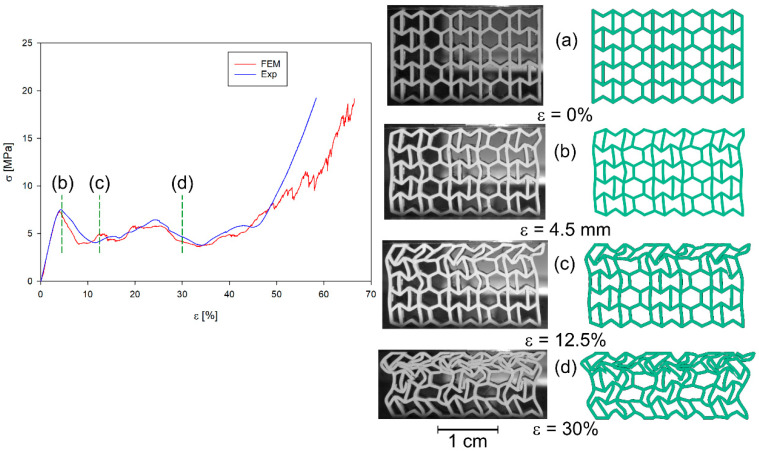
Compression stress–strain curve of experimental and FE analysis results for *b_y_* structure (**left**) and corresponding deformation behaviours at selected compression strains (**right**).

All four structures presented in Figure 1 were also analysed using the finite element method (FEM). The numerical analysis results were compared with experimental data for selected stress–strain curves (Figure 12, Figure 13, Figure 14 and Figure 15). The stiffness of auxetic unit cells is lower than that of hexagonal unit cells, which causes the onset of plastic deformation to occur first in the auxetic cells. The subsequent deformation behaviour is closely related to the positioning of these elements within the studied structure (Figure 12, Figure 13, Figure 14 and Figure 15).

The stiffness of the auxetic basic cells is lower than that of the hexagonal basic cells, so the process of plastic deformation of the elements under consideration starts with the auxetic cells. The further course of deformation is also closely related to the position of these elements in the structure under consideration (Figure 12, Figure 13, Figure 14 and Figure 15).

In all cases analysed, very good agreement was obtained between the results of the numerical analyses and the experimental studies, at least in the initial phase of the compression process. The initial phase of the compression process includes, in addition to the elastic part of the process, the deformation of the structure together with the collapsing/folding of one layer of the structure in question. Similar curve trends have been observed in other areas. It should be noted that much smaller differences between experimental studies and numerical analyses occur in the representation of the behaviour of b-type structures (Figure 14 and Figure 15) than of a-type structures (Figure 12 and Figure 13). This is related to the representation of the properties of the structures. It is evident from the stress–strain curves that, for b-type structures, bending-dominated deformation plays a major role in the deformation process of the structure. Structures in which bending plays a dominant role show higher energy absorption capacities [52]. In contrast, in the case of *a_x_* and *a_y_* structures, stretching dominates the folding of structures [19,52]. When the behaviour of the structures is determined by axial forces, then the material failure process has by far the greater influence on the stress–strain curves. The FE model assumes that the material is homogeneous. However, studies of 3D-printed specimens made of both thermoplastic materials and metal alloys show that they often do not have a homogeneous structure [53]. Hence, in the case of experimental studies, the course of the maxima and minima of the stress–strain curves are smoother compared to the results of numerical analyses. The differences that occur are also influenced by the finite size of the finite elements used in the simulation studies.

**Figure 16:**
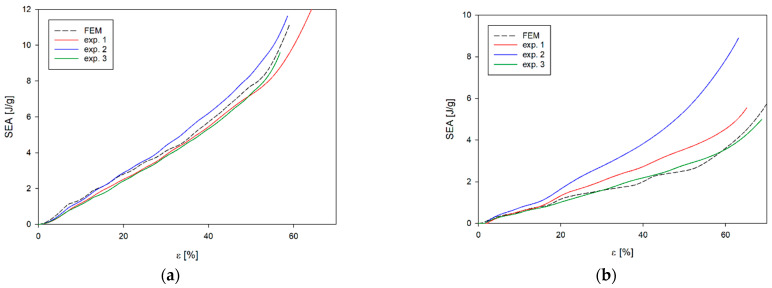
Compression SEA curve of experimental and FE analysis results for (**a**) *a_x_* structure and (**b**) *a_y_* structure.

**Figure 17:**
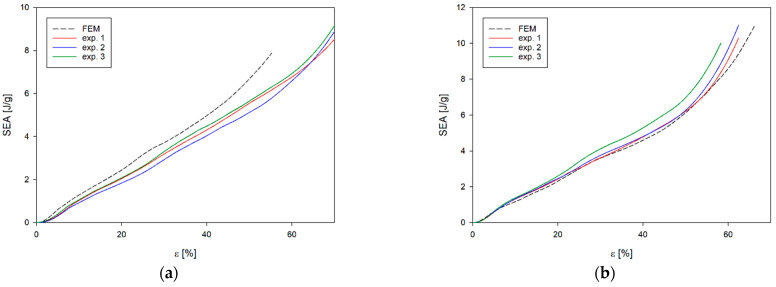
Compression SEA curve of experimental and FE analysis results for (**a**) *b_x_* structure and (**b**) *b_y_* structure.

**Figure 18:**
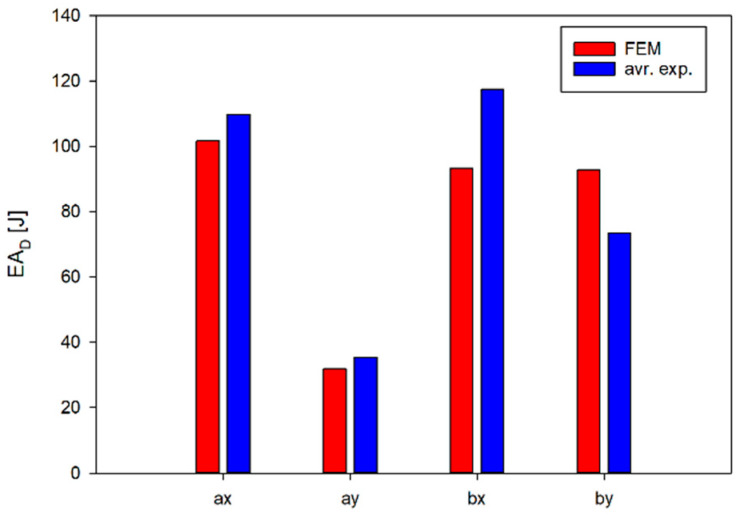
Comparison between the simulation and experimental results of the EA for all of the studied structures.

Although the nature of the changes in EA and SEA obtained from the numerical analyses (Figure 16 and Figure 17) is analogous to the results obtained from experimental studies, the greatest differences are found for b-type structures. In the case of numerical analyses of the loading of *b_x_* and *b_y_* structures, the results obtained are practically not influenced by the direction of loading of the structures; however, the differences are found in experimental studies. This is due to the largest difference between the EA(ε) curve obtained from numerical analyses and experimental tests. In this case, the numerical curve lies above the experimental results (Figure 17a). This situation does not occur in the other cases (Figure 16a,b and Figure 17b). These differences translate into parameters characterising the structures obtained from the numerical analyses (Table 6 and Figure 18).

### 6.2. Parametric Simulation Results

Parametric analyses were carried out of different thicknesses *t* of the structure elements. The difference in thicknesses varied from 0 to 10% in 5% increments in both directions. This translates into a change in relative density of the structure from 24.5 to 30.6%.

**Figure 19:**
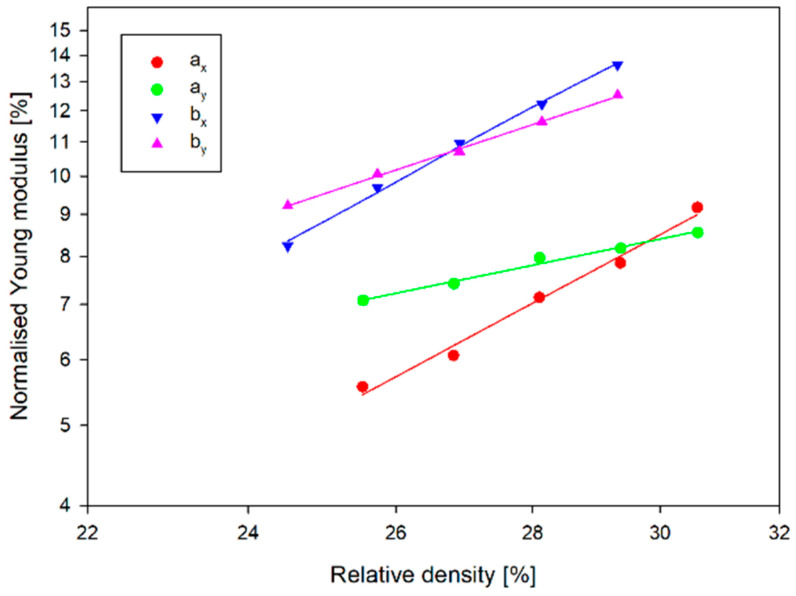
Normalised Young’s modulus vs. relative density.

Figure 19 shows the normalised Young’s modulus vs. relative density. A-type structures, regardless of direction, have a lower Young’s modulus than structures of type b. In contrast, the variation of Young’s modulus as a function of relative density (wall thickness t) is significantly greater for structures *a_x_* and *b_x_* than for the other structures. In the case of the *a_x_* structure, with a 20% change in wall thickness, Young’s modulus increased by more than 120 MPa. In the case of the _ay_ structure, however, it only increased by about 40 MPa.

**Figure 20:**
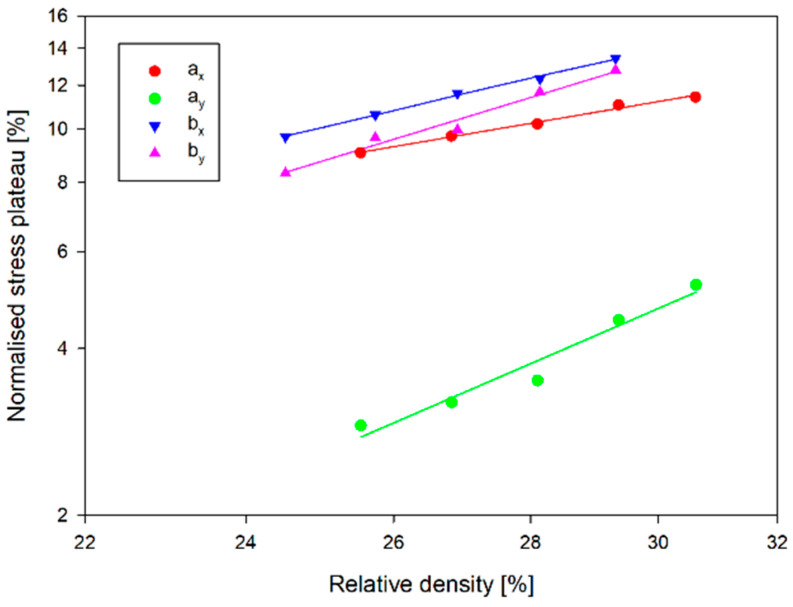
Normalised stress plateau vs. relative density.

**Figure 21:**
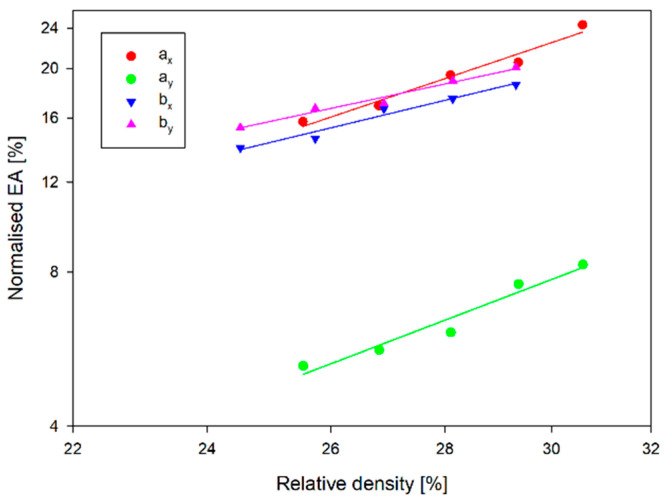
Normalised EA vs. relative density.

**Figure 22:**
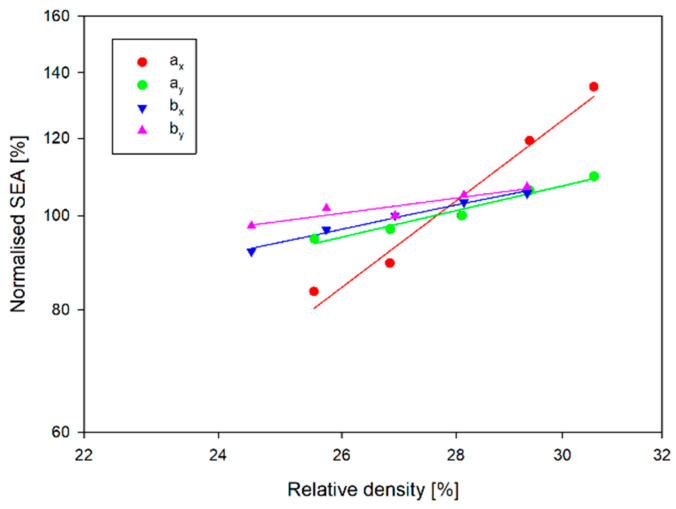
Normalised SEA vs. relative density.

The plateau and EA stress is also more sensitive to a change in relative density for *a_x_* and *b_x_* structures than for the other structures (Figure 20 and Figure 21). The plateau stress increased for structures *a_x_* and *a_y_* by 2.4 MPa and 2.3 MPa, respectively, with a 20% change in wall thickness. In contrast, for *b_x_* and *b_y_* structures, the change was 4.4 MPa and 3.7 MPa, respectively. Note that the plateau stress of the *a_x_* structure for the relative density range analysed is comparable to the plateau stresses for the type-b structures. This translates into EA values for this structure in the considered wall thickness range. For smaller values of the wall thickness of the structures, the EA value of this structure is only inferior to the EA value of the *b_y_* structure. The significant increase in EA with increasing wall thickness means that for a wall thickness equal to *t*, these quantities are comparable, and for the thickest wall, the EA of the *a_x_* structure is 42.3 J greater than that of the *b_y_* structure. The differences that occur are particularly evident in the case of the normalised SEA shown in Figure 22. This figure shows the SEA normalised to the SEA value corresponding to a structure with a wall thickness of *t* and expressed as a percentage, so all straights pass through 100%. A 10% change in wall thickness from the reference value t results in a change in SEA of 5–9% for all structures analysed except the *a_x_* structure. For the *a_x_* structure, the change in SEA is 35.5% in this range, corresponding to almost 2.46 J/g.

Although the structures are made of a thermoplastic material, with Pearson’s coefficient R^2^ > 95%, they confirm the linear agreement of the results with the relationships obtained from cellular theory (7)–(9) in the range analysed [19]. Table 7 contains the determined coefficients (Equations (7)–(9)) characterising not only the normalised Young’s modulus but also the EA and plateau stress. However, the obtained values of the *n* parameters deviate from the analytical values. The value of the exponent *n* of the normalised plateau stress and the normalised Young’s modulus should be less than 1 in the case of stretching-dominated structures, while in the case of bending-dominated structures: n_E_ > 2 and n_pl_ > 1.5 [19]. However, the n_E_ coefficient of the *a_x_* structure is 2.76, with the F − Δl curve having a typically stretching-dominated character (Figure 9). However, closer to the analytical value is the coefficient n_pl_ = 1.31.

## 7. Conclusions

**Figure 23:**
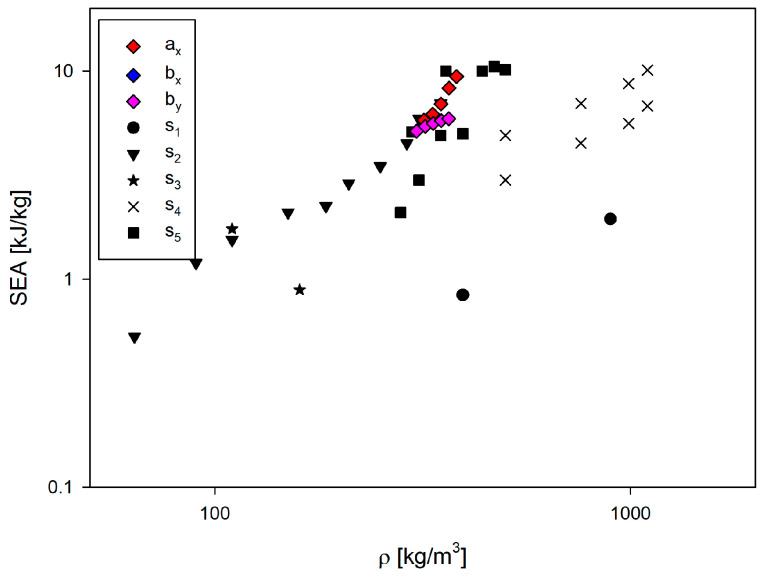
Comparison of SEA under in-plane compression. Additional structures from literature: s_1_ [52], s_2_ [53], s_3_ [55], s_4_ [56] and s_5_ [57].

Based on a combination of hexagonal honeycomb and re-entrant honeycomb cells, the concept of novel hybrid cell structures was developed. Experimental studies and numerical analyses of the behaviour of the analysed structures under in-plane compression in two compression directions were carried out. Explicit finite element analyses with an explicit integration scheme, incorporating plastic deformation and ductile damage evolution models, were employed to analyse the entire deformation process, including plastic and damage stages. Good agreement was obtained between the results of the numerical analyses and the experimental studies. The excellent in-plane specific energy absorption of structures *b_x_*, *b_y_*, and especially *a_x_* is observed in several competitive cellular structures [54,55,56,57,58,59] (Figure 23).

It was shown that the behaviour of the *a_x_* structure deviates from the predictions of cellular theory. Compared to the other structures analysed, the *a_x_* structure is characterised by a large increase in EA with a 20% change in wall thickness and a very large increase in SEA of about 42% (3.6 J/g).

Numerical analyses and experimental studies have shown that the b-type structures have similar characteristic parameters irrespective of the loading direction and are a little less than those characterising the *a_x_* structure. The low mass and lack of corrosion, together with the low sensitivity to the load direction of *b_x_* and *b_y_* structures, prompts their use in protective solutions where we cannot predict the load direction in advance, such as in automotive protective systems.

In addition to this, an alternative approach was proposed for cases where the energy absorption efficiency parameter cannot be used to determine densification strain. The proposed solution made it possible to determine parameters characterising cellular structures without a detailed analysis of the energy absorption efficiency parameter.

## Figures and Tables

**Table 1:** Fixed process parameters of INTAMSYS FUNMAT HT Enhanced.

Nozzle temperature	400 °C
Cooling ratio	50%
Filling ratio	100%
Platform temperature	150 °C
Nozzle diameter	0.4 mm
Printing speed	60 mm/s
Pattern filling	linear
Layer thickness	0.1 mm
Raster orientation	−45°/+45°

**Table 2:** Settings Nikon XT H 225 ST XCT system to scan a ULTEM 9085 cellular AuxHex structures manufactured by HT FFF method.

Material	Tungsten
Source voltage	120 kV
Source power	16 W
Exposure time	1500 ms
Filter	No
Continuous rotation	Yes

**Table 3:** Dimensional accuracy of produced samples.

	* 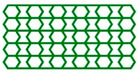 * *a_x_*	* 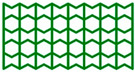 * *a_y_*	* 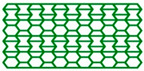 * *b_x_*	* 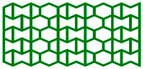 * *b_y_*
Length [mm]	66.68 ± 0.17 (67)	56.72 ± 0.11 (57)	69.74 ± 0.15 (69.9)	53.32 ± 0.12 (53.5)
Width [mm]	35.74 ± 0.18 (36)	31.52 ± 0.10 (31.8)	42.74 ± 0.12 (43)	33.82 ± 0.13 (34.1)
Height [mm]	19.82 ± 0.12 (20)	19.86 ± 0.14 (20)	19.84 ± 0.11 (20)	19.83 ± 0.12 (20)
Wall thickness [mm]	1.05 ± 0.07 (1)	1.05 ± 0.07 (1)	1.05 ± 0.05 (1)	1.05 ± 0.04 (1)

**Table 4:** ULTEM 9085 material parameters.

Experiment No.	Young’s Modulus [MPa]	Strain at Fracture [-]	Maximum Stress [MPa]	Maximum Load Force [kN]	Strain Rate [1/s]
1	2783	0.0569	81.29	1.16	0.0033
2	2496	0.0633	78.64	1.11	0.0033
3	2593	0.0625	85.19	1.14	0.0033
Average	2624	0.061	81.7	1.14	0.0033
Std. dev.	119.20	0.0028	2.6902	0.0205	-

**Table 5:** Structure parameters.

	Experiment No.		*a_x_*	*a_y_*	*b_x_*	*b_y_*
σ*_fps_* [MPa]	1		6.61	4.82	7.65	6.40
	2		8.38	5.62	8.34	6.61
	3		8.27	4.04	9.28	7.53
Average			7.75	4.83	8.42	6.85
Std. deviation			0.99	0.79	0.82	0.6
*E_c_* [MPa]	1		162.4	220.8	179.4	195.9
	2		194.8	270.4	175.7	201.3
	3		172.2	197.6	203.1	221.1
Average			176.5	229.6	186.1	206.1
Std. deviation			16.6	37.2	14.9	13.3
*ε_Y_*	1		0.0291	0.0213	0.0489	0.0322
	2		0.0336	0.0214	0.0365	0.0315
	3		0.0295	0.0216	0.0369	0.0328
Average			0.0307	0.0214	0.0408	0.0322
Std. deviation			0.0025	0.0002	0.0071	0.00065
*ε_D_*		1	0.466	0.427	0.629	0.506
		2	0.566	0.326	0.536	0.474
		3	0.448	0.549	0.548	0.433
Average			0.49	0.434	0.571	0.471
Std. deviation			0.06	0.11	0.051	0.036
σ*_C pl_* [MPa]		1	7.00	2.73	5.97	4.46
		2	6.81	3.58	6.29	4.80
		3	5.98	2.15	7.00	5.17
Average			6.60	2.82	6.42	4.81
Std. deviation			0.54	0.72	0.53	0.36
*EA_D_* [J]		1	133.2	34.69	120.9	73.7
		2	92.3	35.05	107.8	73.9
		3	103.9	36.34	123.9	72.9
Average			109.8	35.36	117.5	73.5
Std. deviation			21.1	0.87	8.56	0.53
*SEA_D_* [J/g]		1	7.59	2.97	7.19	6.33
		2	8.98	3.00	5.55	6.29
		3	6.22	3.11	6.25	5.78
Average			7.60	3.03	6.33	6.13
Std. deviation			1.38	0.07	0.83	0.31

**Table 6:** Numerical structure parameters.

	*a_x_*	*a_y_*	*b_x_*	*b_y_*
σ*_fps_* [MPa]	7.94	4.12	8.48	7.28
*E_c_* [MPa]	197.24	209.1	206.8	225.11
*ε_Y_*	0.021	0.014	0.023	0.022
*ε_D_*	0.46	0.56	0.46	0.51
σ*_C pl_* [MPa]	5.75	1.84	6.15	5.20
*EA_D_* [J]	101.7	31.9	93.3	92.8
*SEA_D_* [J/g]	8.24	3.07	5.97	5.65

**Table 7:** Parameters *n* and *a* from Equations (7)–(9) determined for the analysed structures.

	a_E_	n_E_	a_pl_	n_pl_	a_EA_	n_EA_
*a_x_*	7.1 × 10^−4^	2.76	0.13	1.31	0.08	2.34
*a_y_*	0.23	1.06	5.76 × 10^−5^	3.33	9.4 × 10^−3^	2.65
*b_x_*	1.2× 10^−3^	2.78	0.03	1.8	0.66	1.67
*b_y_*	0.04	1.7	4.6× 10^−3^	2.35	1.31	1.49

## Data Availability

The raw data supporting the conclusions of this article will be made available by the authors on request. The data are not publicly available due to the University data sharing policy.
